# Impact of adsorbate–substrate interaction on nanostructured thin films growth during low-pressure condensation

**DOI:** 10.3762/bjnano.16.36

**Published:** 2025-03-28

**Authors:** Alina V Dvornichenko, Vasyl O Kharchenko, Dmitrii O Kharchenko

**Affiliations:** 1 Sumy State University, 116 Kharkivska St., 40007 Sumy, Ukraine; 2 Institute of Applied Physics, National Academy of Sciences of Ukraine, 58 Petropavlivska St., 40000 Sumy, Ukraine

**Keywords:** adsorbate–substrate interaction, adsorptive systems, numerical simulations, pattern formation

## Abstract

We discuss effects of elastic adsorbate–substrate interactions in processes of nanostructuring of thin films during low-pressure condensation in the framework of theoretical approaches and numerical simulations. It will be shown that an increase in the elastic interaction strength induces first-order transitions and pattern formation. We simulate deposition on one- and multicomponent substrates with different strengths of adsorbate–substrate interactions. We will show that an increase in the strength of adsorbate–substrate interactions stimulates the formation of stable surface structures during deposition, which leads to an increase in its coverage and the formation of a smaller number of adsorbate islands of larger size. At elevated adsorption rates, an increase in adsorbate–substrate interactions results in the transformation of the surface morphology and the formation of percolating adsorbate structures. Deposition onto multicomponent substrates leads to the formation of a stationary surface morphology with an elevated number of adsorbate islands of smaller size, compared to one-component substrates. This study provides a deep insight into the peculiarities of nanostructured thin films’ growth in low-pressure systems with different adsorbate–substrate bonding.

## Introduction

Innovative nanostructured thin films are widely exploited in ground-breaking developments regarding transistors [1–2], energy harvesting [3–4], sensors [5], and catalysts [6–8]. Nanostructured thin films grown via low-pressure deposition methods have garnered significant attention because of their diverse applications in electronics, optics, catalysis, and sensors [9]. The ability to precisely control properties such as morphology, crystallinity, and surface chemistry of the films is crucial for optimizing performance in these applications. In adsorption–desorption processes, where materials are deposited from the gas phase, experimental techniques enable the study the formation of clusters or islands of adsorbed molecules/atoms, which can have nanometer-scale linear dimensions [10–11]. Experimental observations showed that nanometer-scale vacancy islands can arrange into a perfect triangular lattice when a single monolayer of Ag is deposited on a Ru(0001) surface at room temperature [12]. Similar elongated nanometer-sized islands were observed for Si/Si(100) [13], as well as during Ge deposition on Si [14]. Additionally, elongated metallic islands were detected during the deposition of Cu on Pd(110) [15] and Ag on Cu(110) [16]. Small separated adsorbate islands of Al and Cu condensates were obtained on glass substrates [17–19]. Hence, the usage of different species adsorbed on different substrates results in various surface patterns and defines the morphology of the growing surface. Such patterns emerge from the interplay of quasi-chemical reactions, lateral interactions between the adsorbed particles, and adsorbate–substrate interactions on scales shorter than the diffusion length. The strength of adsorbate–substrate interaction is defined by both substrate and adsorbed material.

Adsorbate–substrate interactions encompass a broad spectrum of physical and chemical phenomena that dictate the initial nucleation, subsequent growth kinetics, and final structural properties of thin films. These interactions are influenced by factors such as surface energetics, lattice matching, van der Waals forces, and chemical bonding configurations [20–21]. Strong interactions can lead to ordered nucleation and the formation of well-defined islands or clusters. The strength and nature of these interactions play a pivotal role in determining whether the film growth follows layer-by-layer (Frank–van der Merwe) or island (Volmer–Weber) growth modes [22]. Therefore, central to achieving the control the type and size of surface patterns is understanding how adsorbate–substrate interactions influence the growth dynamics of these films [23–25].

Mathematical and numerical modeling of the growth processes of nanostructured thin films enables detailed analysis of the dynamics involved. It allows for investigating the impact of fundamental factors such as chamber pressure, deposition temperature, energy characteristics, and external influences on the morphology, type, and size of surface structures during growth. A widely adopted approach for mathematical modeling in this context is based on reaction–diffusion models [26–34]. This method typically provides insights that can guide adjustments to the technological parameters to achieve thin films with desired physical and chemical properties. Here, the competition between atomic deposition and reactions may stabilize nanometer-scale spatial patterns, even for monoatomic layers [34]. The patterns may serve as templates for the later evolution of film textures. Relevant examples of such systems are Al or Cu layers deposited on Si substrates, or SiO_2_ and TiN layers deposited on Ti or Al substrates [31].

In this article, we perform a computational study of the evolution of a monoatomic layer deposited on a substrate during low-pressure condensation in the framework of a continuous dynamical model of the reaction–diffusion type. This approach allows one to model deposition techniques such as low-pressure CVD (LPCVD) at sub-atmospheric pressures [35]. We discuss effects of adsorbate–substrate interactions in dynamics of adsorbate structures formation and their statistical properties. It will be shown, that an increase in the strength of adsorbate–substrate interactions can induce adsorbate patterning on the first growing layer and lead to morphological transformation of the growing layer. The results obtained within this work can be used to control the morphology of the growing thin films, that is, type and size of surface structures, by exploiting different deposited materials on different substrates.

The paper is organized in the following manner. In the section “Model”, we derive the one-layer model of reaction–diffusion type for the spatio-temporal evolution of adsorbate concentration on the substrate. In the section “Results and Discussion”, we initially discuss first-order transitions induced by the strength of adsorbate–substrate interactions in a homogeneous system. Next, we perform stability analysis and define a range of the strength of adsorbate–substrate interaction responsible for pattern formation during deposition. Finally, we present results of numerical simulations and discuss dynamics of surface patterns formation and statistical properties of the surface structures. We conclude in the last section.

## Model

We consider the two-layer model describing the spatio-temporal evolution of adsorbate on the substrate during condensation in adsorptive systems. We assume deposition of atoms of one type and exploit the formalism of reaction–diffusion models to monitor the local concentration of adsorbate *x*_1_(**r**,*t*) ∈ [0,1] in a unit cell with size ℓ on the first growing layer. We follow an approach discussed in [36] and assume the concentration on the second layer in a form


[1] $$x_{2}\left(x_{1}\right)={\left(\sqrt{x_{1}}-\beta \right)}^{2},$$

where β is the dimensionless terrace width of multilayer structures.

In the general case, the reaction–diffusion equation for the field *x*_1_(**r**,*t*) has the form


[2] $$\frac{\partial x_{1}\left(r,t\right)}{\partial t}=R\left(x_{1},x_{2}\right)-\nabla \cdot J\left(x_{1}\right)+\xi \left(r,t\right).$$

The reaction term *R*(*x*_1_,*x*_2_) is responsible for adsorption, desorption, and diffusion of adatoms between neighbor layers. The adsorbate flow **J** is defined through the free energy ℱ of the adsorbed layer


[3]

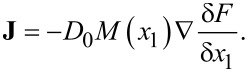



Here, *D*_0_ is the diffusion coefficient; *M* = *x*_1_(1 − *x*_1_) takes into account that diffusion is possible onto free sites; ∇ = d/d**r**. The last term in Equation 2 represents a stochastic source that takes into account the effects of redistribution of adatoms at the microscopic level, describing the system at the mesoscopic level. In the simplest case, we choose it as white zero-mean delta-correlated Gaussian noise: ⟨ξ(**r**,*t*)⟩ = 0, ⟨ξ(**r**,*t*)ξ(**r’**,t’)⟩ = σ^2^δ(**r**− **r’**)(*t* − *t*’) with the intensity σ^2^.

The reaction term for the first adsorptive layer has the following form:


[4] $$R\left(x_{1}\right)=R_{a}\left(x_{1}\right)+R_{d}\left(x_{1}\right)+R_{t}\left(x_{1}\right).$$

Adsorption processes are described by the term *R_a_* = *k_a_*(1 − *x*_1_)(1 − *x*_2_), where the adsorption rate *k_a_* = ω*p*exp(−*E_a_*/*k*_B_*T*) is defined through the adsorption energy *E_a_*, the frequency factor ω, and the pressure of the gaseous phase *p*; *k*_B_ is the Boltzmann constant; *T* is the temperature. They require free sites on both first (1 − *x*_1_) and second (1 − *x*_2_) layers. The desorption term *R_d_* = −*k_d_x*_1_(1 − *x*_2_)exp(*U*(*x*_1_)/*k*_B_*T*) is defined by the desorption rate *k_d_* = ωexp(−*E_d_*/*k_B_T*), where *E_d_* is the desorption energy, and the interaction potential *U*(*x*_1_), including adsorbate–adsorbate and elastic adsorbate–substrate interactions. Transference reactions between layers describe a decrease in the adsorbate concentration on the current layer by the term *R_t_* = *k_t_*(*x*_2_ − *x*_1_).

The free energy ℱ in Equation 3 for the first adsorptive layer has the form [31–32]:


[5]

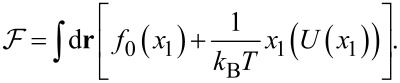



The first term on the right-hand side of Equation 5 is responsible for the entropic non-interactive part, *f*_0_(*x*_1_) = *x*_1_ln(*x*_1_) + (1 − *x*_1_)ln(1 − *x*_1_). The interaction potential *U*(*x*_1_) includes the term describing interaction between substrate and adsorbed particles *U_el_* = 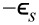 with the strength 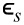 *>* 0 (attractive interaction) and interactions between adsorbed particles described by the interaction potential


[6] $$U_{ads}\left(r\right)=-\int \text{d}r^{\prime }\left[u\left(r-r^{\prime }\right)x\left(r^{\prime }\right)\right].$$

It is defined through the binary attractive potential −*u*(*r*) of symmetrical form, 
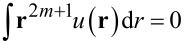
, *m* = 1,…,∞. Following [30–32], we choose the Gaussian profile as a simple approximation for the interaction potential:


[7]

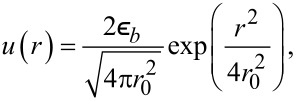



where 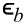 is the interaction strength and *r*_0_ is the interaction radius. Assuming that the adsorbate concentration does not vary significantly within the interaction radius, we can exploit a self-consistent approximation used in different areas of numerical modeling [33–34,36–39]:


[8] $$\int u\left(r-r^{\prime }\right)x\left(r^{\prime }\right)\text{d}r^{\prime }≃\int u\left(r-r^{\prime }\right)\sum_{m}\frac{{\left(r-r^{\prime }\right)}^{m}}{m!}\nabla^{m}x\left(r\right)\text{d}r^{\prime }.$$

Substituting Equation 7 into Equation 8 and assuming 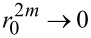 at *m* ≥ 2, the interaction potential of the adsorbate *U_ads_*(**r**) obtains the following form: *U_ads_*(**r**) = 
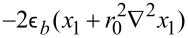
.

It should be noted that the term *U_el_* is important when the bonding between adatoms and substrate is strong (for example Cu atoms deposited onto Ti, Ta, or Mo substrates) [30–31]. For sufficiently small lattice mismatch between adsorbate and substrate, elasticity and stress effects may be neglected (e.g., during deposition of Al atoms on TiN surfaces, the lattice mismatch is about 4%) [40]. From Equation 3, it follows that these interactions do not affect the lateral flux *J* ∝ ∇*U*. At the same time, for systems with strong lattice mismatch, strong adatom–substrate bonding makes desorption negligible during the growth of the first layer because the desorption rate is defined by the interaction potential *U*(*x*_1_).

Next, it is more convenient to move to dimensionless constants α = *k_a_*/*k_d_*, γ = *k_t_*/*k_d_*, by scaling time in units of lifetime of adatoms 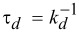. We scale the spatial coordinate **r** in units of ℓ and introduce the diffusion length 
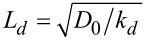
 by using the relations *L_d_* = 20*r*_0_ and ℓ = 4*r*_0_. By combining all reaction terms and diffusion parts, Equation 2 for the spatio-temporal evolution of adsorbate concentration *x* ≡ *x*_1_ on the first growing layer during condensation becomes


[9]

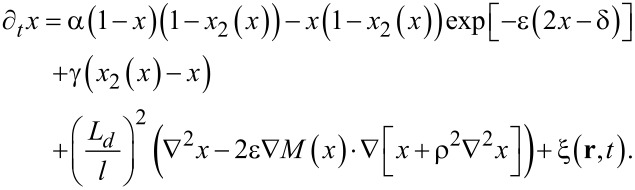



Here, we put 
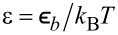
 and 
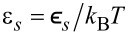
. We also introduce δ = ε*_s_*/ε and ρ = *r*_0_/ℓ. In previous studies it was shown that in the framework of the reaction–diffusion modeling, the surface morphology can be controlled by different factors. For instance, an increase in the adsorption rate leads to an increase in the linear size of separated nanodots on the surface, the formation of percolating structures of adsorbate, and the formation of nanoholes inside the adsorbate matrix [33]. An increase in the adsorbate–adsorbate interaction strength leads to formation of elevated numbers of small adsorbate structures [33,37]. An increase in the rate of transference reactions of adatoms between neighbor layers acts in the opposite manner to the adsorption coefficient [41]. An applied external electric field can affect the type and size of the surface structures [37,42–43]. Anisotropy of surface diffusion, induced by properties of the substrate, can lead to fractal structures of the adsorbate [44]. In this work, we concentrate our attention onto effects related to reduced interaction strength δ onto structuring kinetics of thin films during deposition and the morphology of the first growing layer. The interaction strength 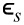 for the specific choice of substrate and adsorbate can be found in real experiments or can be calculated by atomistic simulations (ab initio techniques or molecular dynamics simulations). In our study, a variation in 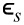 means a usage of different materials for both substrate and adsorbate to simulate the formation of stable surface structures during deposition.

## Results and Discussion

### First-order transitions in a homogeneous system

First, let us discuss homogeneous states *x_st_* and analyze their stability by setting ∇**J** = 0 in Equation 2. Stationary states *x_st_* correspond to ∂*_t_x* = 0 giving *R*(*x*) = 0 with


[10] $$\begin{array}{c} R\left(x\right)=\alpha \left(1-x\right)\left(1-x_{2}\left(x\right)\right)\\ -x\left(1-x_{2}\left(x\right)\right)\exp\left[-\varepsilon \left(2x+\delta \right)\right]+\gamma \left(x_{2}\left(x\right)-x\right). \end{array}$$

**Figure 1:**
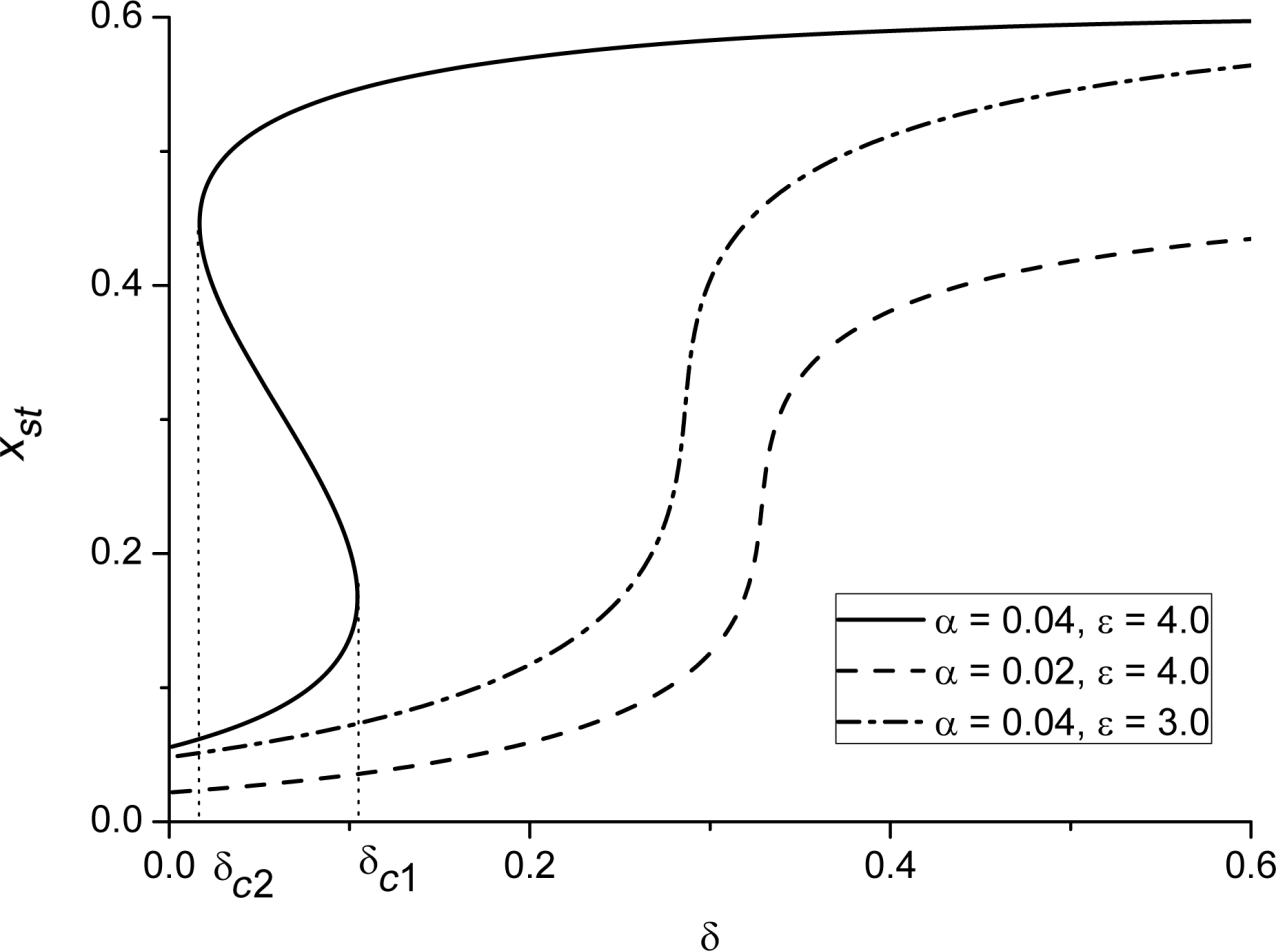
Dependencies of the homogeneous stationary states *x_st_* on the substrate–adsorbate interaction strength δ at different α end ε.

Dependencies of the homogeneous stationary states *x_st_* on the substrate–adsorbate interaction strength δ at different adsorption rates α and adsorbate–adsorbate interaction strengths ε are shown in Figure 1.

It follows that an increase in the interaction strength δ from zero results in first-order transitions from a low-density state towards a high-density state at δ = δ*_c_*_1_ (see solid curve in Figure 1. A decrease in δ leads to the reverse transition at δ = δ*_c_*_2_. By comparing curves in Figure 1, one finds that an increase in either adsorption rate α or adatom interaction strength ε induces these transitions. The critical values δ*_c_*_1_*_,c_*_2_ depend on both α and ε.

**Figure 2:**
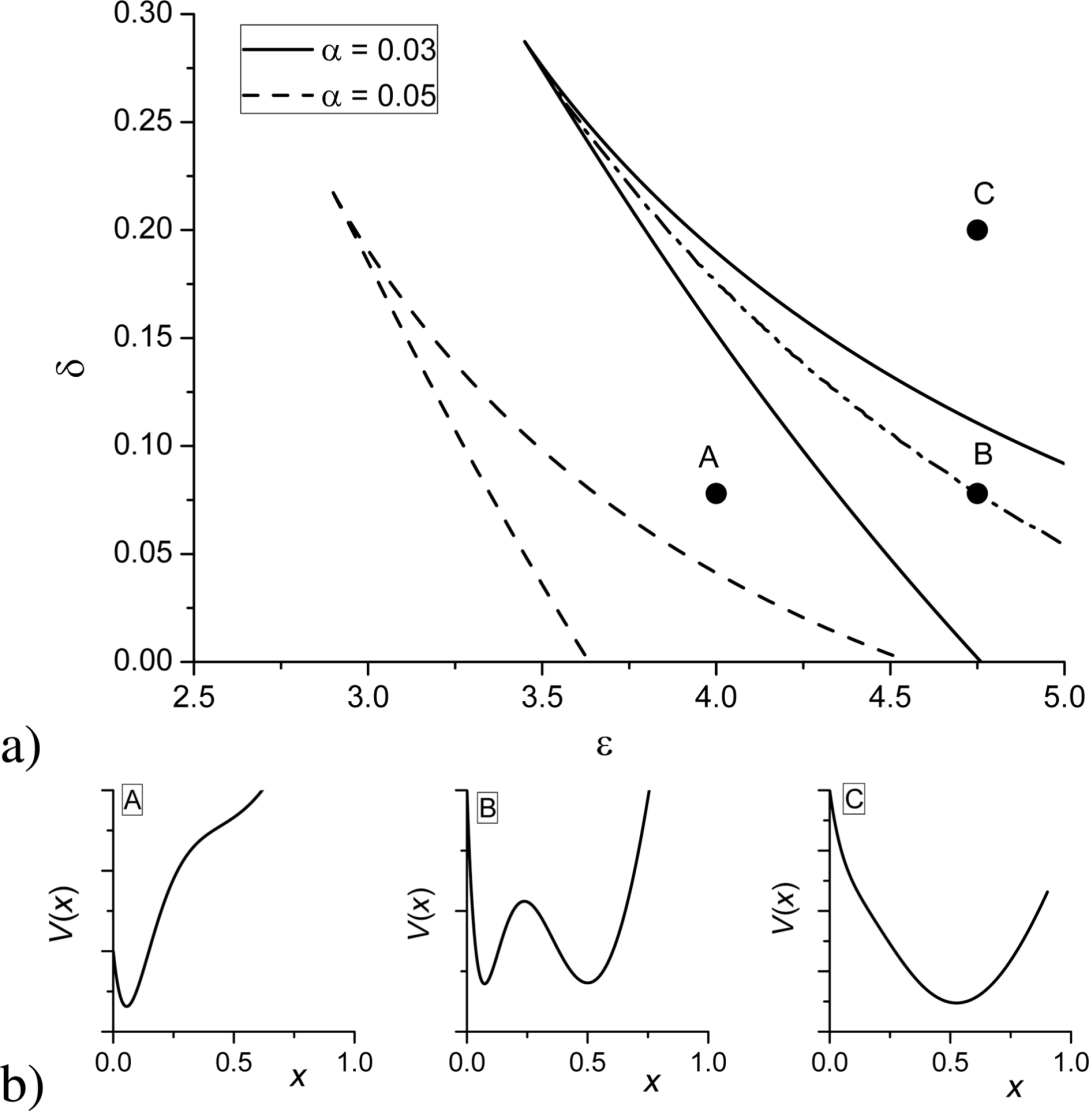
Stability diagram for the first-order transitions at different (a) α and (b) potential *V*(*x*) at different points of the stability diagram at α = 0.03.

By analysis of the dependencies *x_st_*(δ) for different ε, one can obtain the stability diagram δ(ε) at different α, shown in Figure 2a. Let us consider the case α = 0.03. It follows that the bistability domain (between the solid curves) is bounded by the critical values of ε for the fixed strength δ and by the maximal value of the interaction strength δ. Inside this domain, there are three stationary states of the adsorbate concentration (see the solid curve in Figure 1. The dash-dot curve corresponds to the spinodal when the two stable states are equally probable. It can be found by calculating the effective potential *V*(*x*) related to the reaction force Equation 10 as 
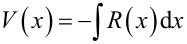
. The form of the potential *V*(*x*) at different points of the stability diagram at α = 0.03 is shown in Figure 2b. From Figure 2a, one finds that an increase in the adsorption rate α requires smaller values of the interaction strength ε for the first-order transitions.

### Stability analysis of spatially extended system

**Figure 3:**
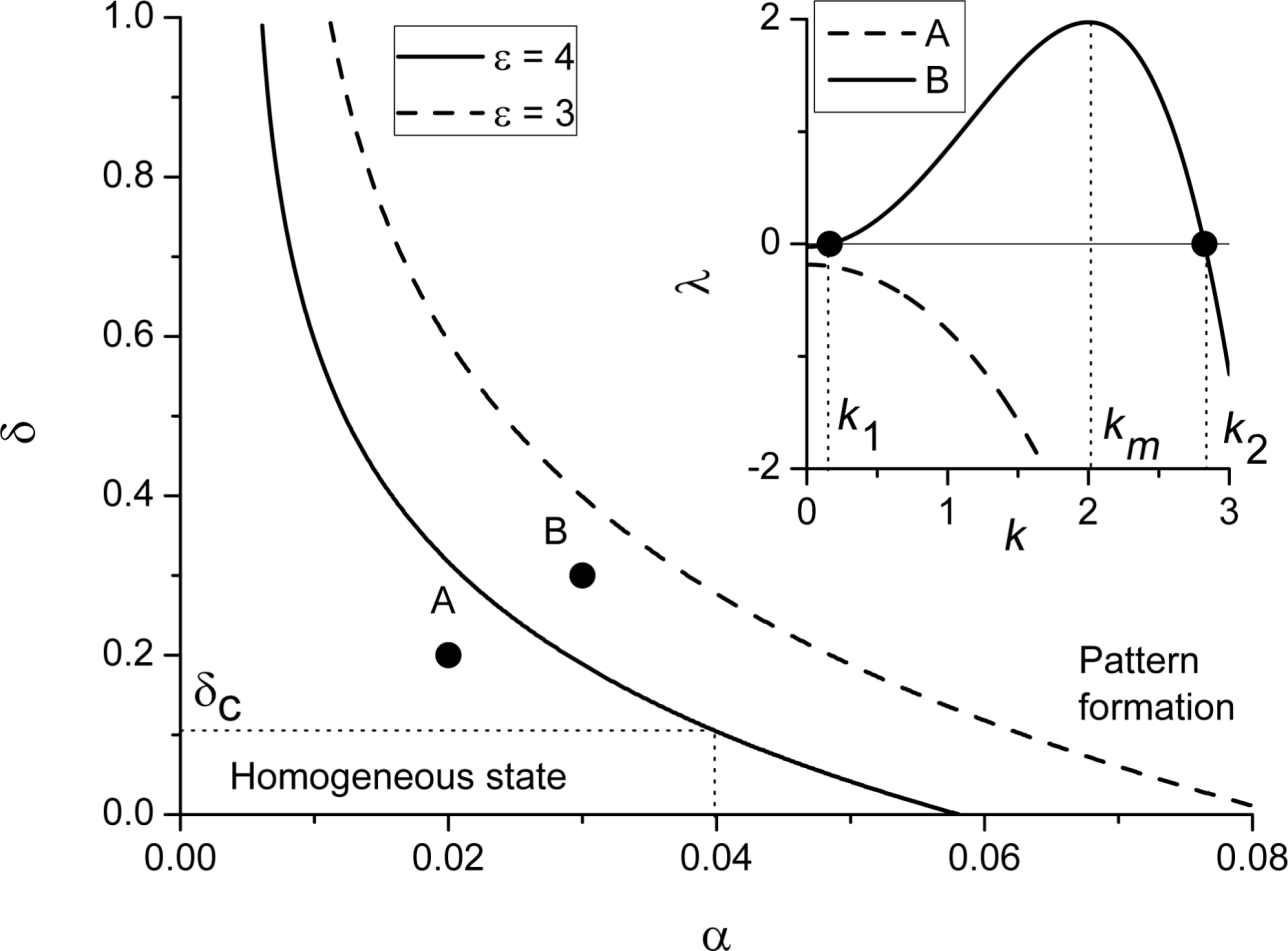
Stability diagram of the stationary homogeneous states to inhomogeneous perturbations at different values of adatom interaction strength ε.

In order to define a role of the adsorbate–substrate interactions characterized by the strength δ in the processes of nanostructured thin films’ growth and its effect onto surface morphology, we first define the conditions of the formation of stable adsorbate structures on the substrate during deposition. To this end, we exploit the standard stability analysis of the stable stationary homogeneous states *x_st_* to inhomogeneous perturbations. To proceed, we consider the small deviation of the adsorbate concentration *x*(**r**) from the homogeneous state *x_st_*, 
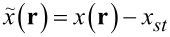
, in the form 
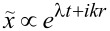
, where *k* is the wave number and λ(*k*) is the stability exponent. This gives 
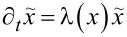
 and 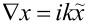. Next, in the framework of linear stability analysis, we expand the reaction term *R*(*x*) in the vicinity of *x_st_* assuming 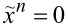 at *n >* 1. From Equation 9, we get the expression for the stability exponent λ(*k*) in the form:


[11] $$\lambda \left(k\right)={{\text{d}}_{x}R\left(x\right)|}_{x=x_{st}}-\kappa k^{2}\left[1-2\varepsilon \mu \left(x_{st}\right)\left(1-\rho^{2}k^{2}\right)\right],$$

where κ = (*L_d_*/ℓ)^2^. The case 
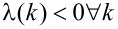
 results in 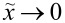 and the system moves to the stable homogeneous state *x_st_*. It means that during deposition, adsorbate will cover the substrate homogeneously, and no separate patterns can be realized. In the case λ(*k*) *>* 0 at *k* ∈ (*k*_1_,*k*_2_) the spatial instabilities will grow over time leading to the formation of stable surface patterns on the substrate. By analyzing the dependencies λ(*k*) for different values of the control parameters, one can define their range when λ(*k*) *>* 0. It was shown previously that during high-pressure condensation, an increase in the adsorption rate leads to pattern formation and change of the surface morphology [33]. We will focus onto the ability of elastic interactions between adsorbate and substrate to induce patterning at low-pressure condensation. The corresponding stability diagram δ(α) illustrating the domain of pattern formation is shown in Figure 3.

It follows that at small adsorption rates (i.e., low-pressure condensation), an increase in the adsorbate–substrate interaction strength δ induces the formation of stable surface structures on the substrate during condensation at δ = δ*_c_*. The typical dependencies of the stability exponent λ on the wave number *k* in domains of homogeneous state (point A) and pattern formation (point B) at ε = 4 are shown in the inset. Here, the most unstable mode *k_m_* defines the mean period of spatial instabilities and can be associated with the inverse mean distance *l* between adsorbate structures, that is, *k_m_* = 2π/*l*. By comparing curves in Figure 3, one finds that a decrease in the adsorbate–adsorbate interaction strength ε requires elevated values of both the elastic interaction strength δ and adsorption rate α for the formation of stable surface patterns during condensation.

### Numerical simulations

In order to monitor the dynamics of adsorbate island formation on the first growing layer, we perform numerical simulations. To proceed, we solve Equation 9 on a two-dimensional hexagonal grid with the linear size *L* = *N*Δ*x* with *N* = 512 sites of the length Δ*x* = 0.5, that is, the spatial integration step; Δ*t* = 10^−3^ is the time step. The Fourier spectral method is used [45–46] to take spatial derivatives. As initial conditions, we assume that the substrate is free of adsorbates by taking *x*(**r**,0) = 0; boundary conditions are periodic. We fix σ^2^ = 0.01 for all simulations.

We exploit the mean adsorbate concentration (coverage) on the substrate ⟨*x*⟩ and the dispersion η = ⟨*x*^2^⟩ − ⟨*x*⟩^2^ to characterize the dynamics of surface morphology change. For spatially extended systems, the dispersion η is an order parameter for pattern formation: η = 0 means that adsorbate homogeneously covers the substrate; a growth of η(*t*) indicates ordering of the coverage field accompanied by the formation of spatial patterns on the substrate. If η(*t*) attains a stationary value η*_st_* ≠ 0, then ordering processes are finished and one has a stable morphology of the layer.

**Figure 4:**
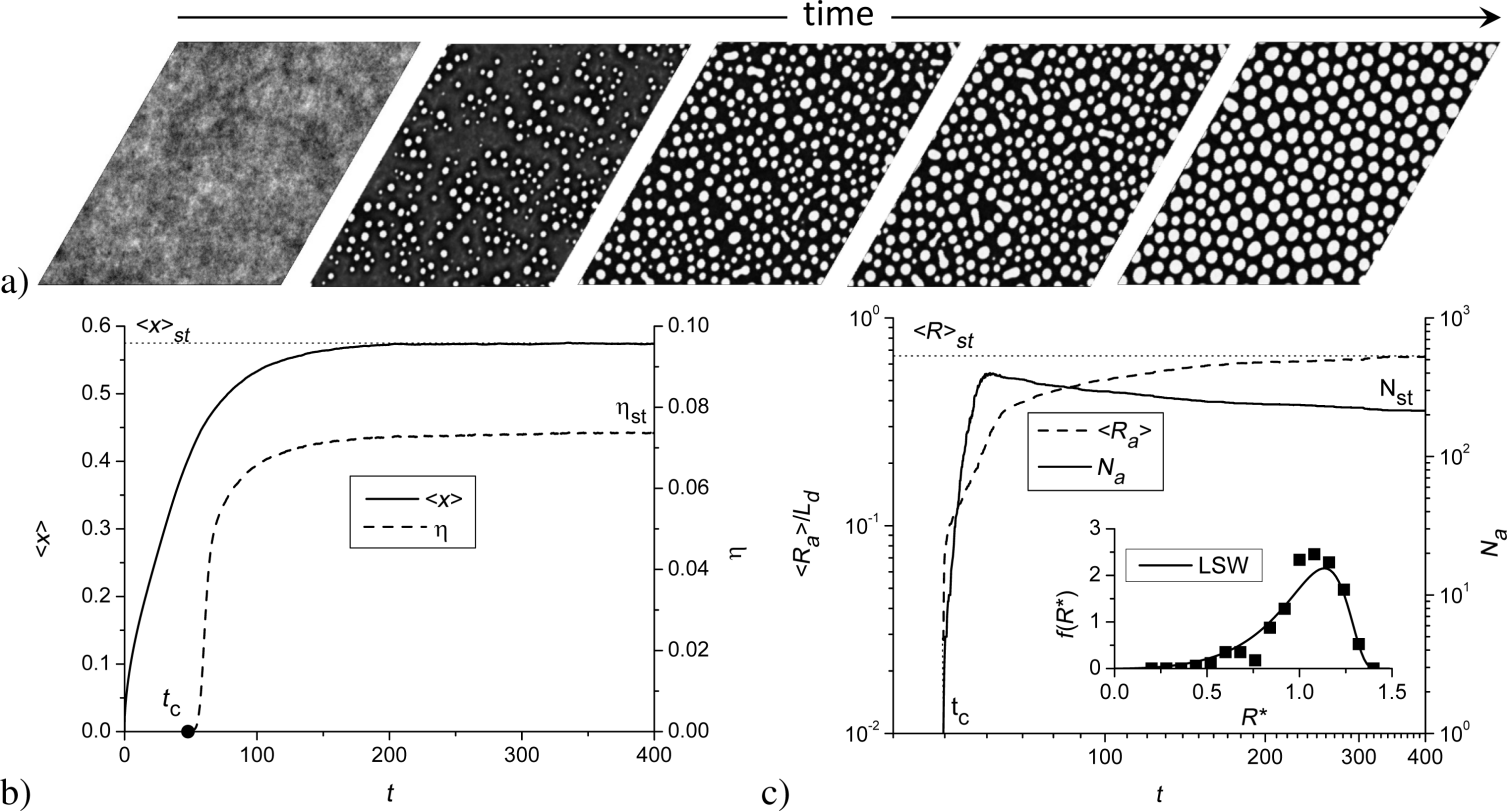
Typical evolution of (a) the morphology of surface of the thin film, (b) the mean adsorbate concentration on the substrate ⟨*x*⟩, and (c) the order parameter η (the inset represents the distribution of islands over sizes R^*^; the curve corresponds to the Lifshitz–Slyozov–Wagner distribution) during deposition at α = 0.04, ε = 4.0, and δ = 0.2.

Typical evolution of the local distribution of the adsorbate concentration during condensation is shown in Figure 4a. Here, the adsorbate concentration is presented using a gray scale from black (*x*(**r**) = 0) to white (*x*(**r**) = 1). One sees that during condensation the adsorbate islands start to organize after some incubation period *t_c_*; with further deposition, the number of these islands increases and they become larger in size. The corresponding dependencies ⟨*x*⟩(*t*) and η(*t*) are shown in Figure 4b. It follows that at initial stages, the mean adsorbate concentration grows; it attains a stationary value ⟨*x_st_*⟩ at late stages. The ordering processes on the surface start after *t* = *t_c_*, when the order parameter η starts to grow. At late stages, η attains the stationary value η*_st_*, indicating the formation of a stable spatial distribution of adsorbate on the substrate.

In Figure 4c, we show the evolution of the mean radius of adsorbate structures ⟨*R_a_*⟩ scaled in units of diffusion length *L_d_* and their number *N_a_*. We associate the linear size of adsorbate structures with the radius of the circle of the same area. One finds that after the incubation time *t_c_*, both quantities start to grow rapidly (i.e., growth stage). After that, *N_a_* attains the maximal value and then starts to decrease, and the dynamics of ⟨*R_a_*⟩ growth is slowing down (i.e., coarsening stage). During long-term condensation, the morphology of the adsorbate becomes stationary and is characterized by *N_st_* adsorbate structures with the mean linear size ⟨*R*⟩*_st_*. The inset in Figure 4c shows the distribution of adsorbate islands over sizes R^*^ = *R_a_*/⟨*R_a_*⟩. Here, the solid curve corresponds to the standard Lifshitz–Slyozov–Wagner distribution [47–48], which relates well to the numerical results represented by squares.

In order to perform a comprehensive analysis of the influence of the substrate–adsorbate interaction strength onto pattern formation during deposition, we consider here two different cases. The first one relates to the one-component substrates (OCS) characterized by different values of δ. The second case is the deposition onto multicomponent substrates (MCS) representing high-entropy alloys, which are formed by mixing equal or relatively large proportions of (usually) five elements. In this case, we assume that the interaction strength δ is different for the different alloying elements of the substrate, that is, δ = δ_0_ + *z*(*m*)Δ, where *m* = 1…5 counts for the different types of atoms in the high-entropy alloy and *z*(*m*) = −2,…,2. In the two-dimensional lattice presentation, the interaction strength δ(**r**) with the randomly distributed *z*(*m*) = *z*(**r**) represents the δ-correlated quenched spatial disorder with ⟨δ(**r**)⟩ = δ_0_; Δ is the strength of the spatial disorder. Next, we analyze the influence of the adsorbate–substrate interaction strength, δ for OCS and ⟨δ⟩ for MCS, onto the incubation time *t_c_* for pattern formation, onto stationary values of the mean coverage ⟨*x*⟩*_st_* and the order parameter η*_st_*, onto the morphology of the surface, and onto stationary values of the number of adsorbate structures *N_st_* and their mean size ⟨*R*⟩*_st_*. To this end, we fix ε = 4 and choose two different values of the adsorption rate α = 0.04 and α = 0.06 (see diagram in Figure 3).

**Figure 5:**
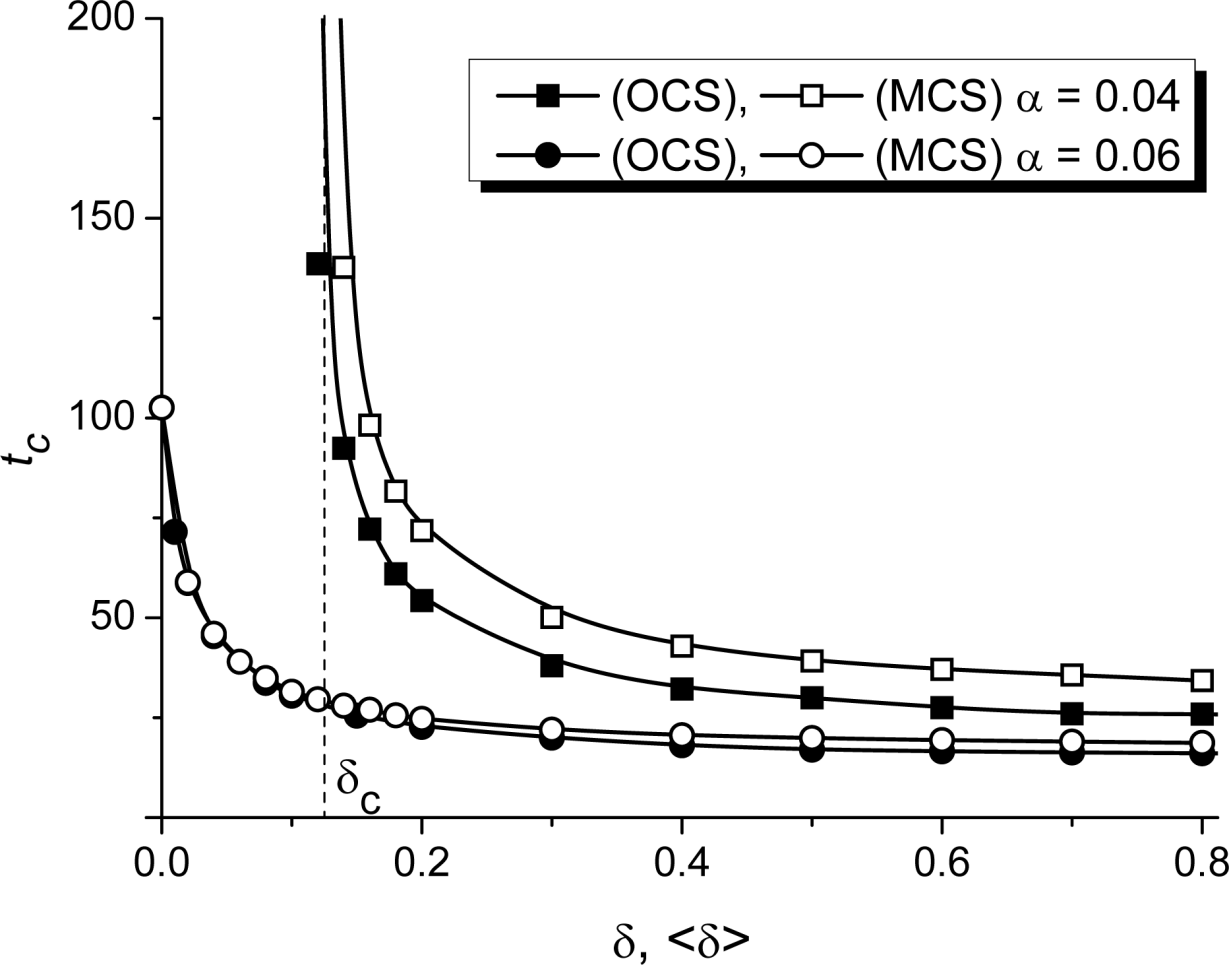
Dependencies of the incubation period *t_c_* on the substrate–adsorbate interaction strength δ at ε = 4.0 and different α for both OCS (filled symbols) and MCS (empty symbols).

First, let us discuss an influence of the elastic interaction strength onto incubation time, shown in Figure 5 for both OCS and MCS, represented by filled and empty symbols, respectively, for different values of α. It follows that for both OCS and MCS, an increase in elastic interaction strength δ leads to a decrease of the incubation time *t_c_* needed for the accumulation of a critical concentration of adsorbate and nucleation of surface structures. For the case of a small adsorption rate α = 0.04, an increase in δ induces these processes at δ = δ*_c_* corresponding well to results obtained in the stability analysis (see Figure 3), verifying the accuracy of the numerical simulations. Moreover, the critical value δ*_c_* does not depend on the structure of the substrate. Here, the structuring of the adsorbate on the OCS is faster than on the MCS (compare filled and empty squares in Figure 5). Hence, at small adsorption rates (i.e., low-pressure deposition) the deposition onto spatially disordered substrates requires enlarged deposition time. At elevated adsorption rates, deposition on both OCS and MCS requires approximately the same time, decreasing with the strength of elastic interactions δ (see filled and empty circles in Figure 5).

**Figure 6:**
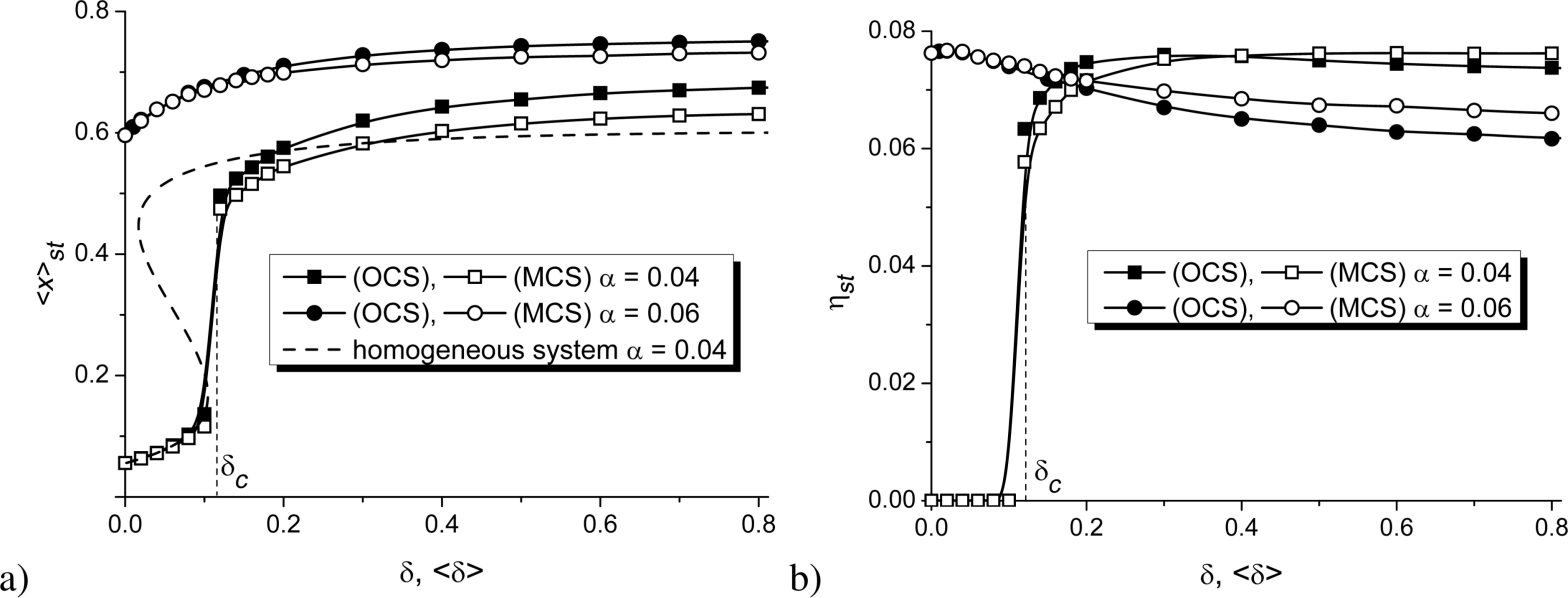
Dependencies of (a) the mean stationary adsorbate concentration ⟨*x*⟩*_st_* and (b) the stationary value of the order parameter η*_st_* on the substrate–adsorbate interaction strength δ at ε = 4.0 and different α for OCS (filled symbols) and MCS (empty symbols).

Next, let us consider the influence of the elastic interaction strength δ onto the stationary coverage of the substrate ⟨*x*⟩*_st_* and the stationary value of the order parameter η*_st_*, shown in Figure 6a,b, respectively for OCS and MCS.

It follows that for the case of elevated α, a growth in δ results in an increase in the substrate coverage and a decrease in the stationary value of the order parameter (see circles in Figure 6). Moreover, the deposition on the OCS leads to an increased concentration of the adsorbate on the substrate that is less ordered compared to the case of MCS (see filled and empty circles in Figure 6a,b). At small α, in the case of weak interactions between adsorbate and substrate, δ *<* δ*_c_*, no stable patterns can be realized, that is, ⟨*x*⟩*_st_* ≪ 1 and η*_st_* = 0 (see filled and empty squares in Figure 6a,b). Moreover, here ⟨*x*⟩*_st_* for the spatially extended system corresponds well to *x_st_* for a homogeneous system (shown by the dashed curve). This result again confirms the accuracy of the numerical simulations. An increase in δ induces ordering processes, and the critical value δ*_c_* corresponds to the δ*_c_*_1_ for the first-order transitions (see solid curve in Figure 1). A further increase in strength of elastic interactions, δ *>* δ*_c_*, leads to an increase in the coverage of the substrate ⟨*x*⟩*_st_*, which is larger for the OCS than for the MCS and does not affect significantly the stationary value of the order parameter η*_st_* for both types of substrates.

**Figure 7:**
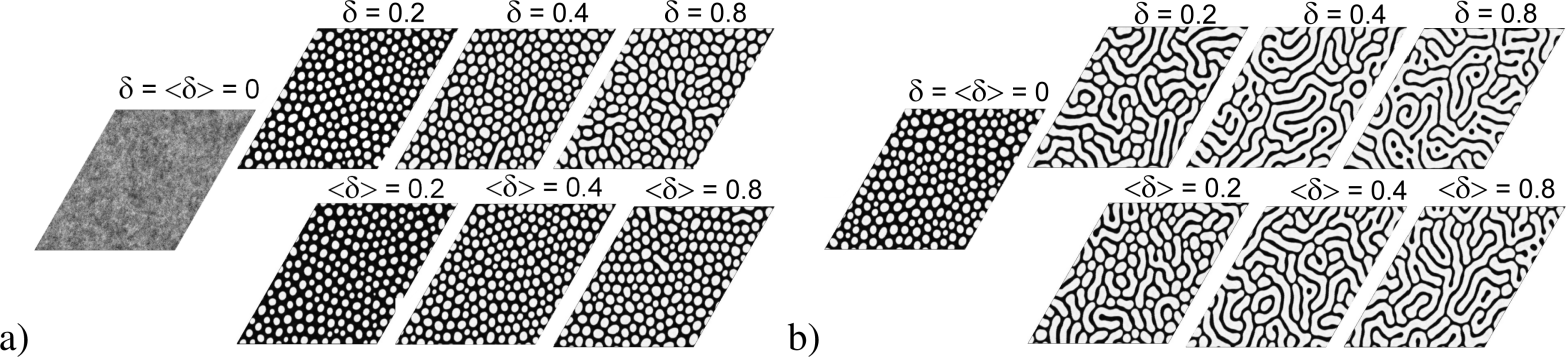
Typical snapshots of the quasi-stationary patterns formed on the OCS (top row) and the MCS (bottom row) at different values of the substrate–adsorbate interaction strength δ at ε = 4.0 and a) α = 0.04; b) α = 0.06.

Next, we discuss a change in the morphology of the adsorptive layer and the statistical properties of surface structures with an increase in elastic interaction strength δ. In Figure 7, we show snapshots of the typical surface morphology realized during deposition at different δ for different values of the adsorption rate α. It is seen that in the case of α = 0.04 (see Figure 7a), without elastic interactions between adsorbate and substrate (δ = ⟨δ⟩ = 0) for both OCS and MCS, one gets a quasi-equilibrium distribution of adsorbate on the substrate without any surface structures. Under such low-pressure deposition conditions, elastic interactions can induce the formation of adsorbate structures on the substrate if δ *>* δ*_c_* with δ*_c_* ≃ 0.105 (see Figure 3). In the last case, one gets separated adsorbate islands (white color) on the substrate during deposition (see snapshots at δ = ⟨δ⟩ = 0.2 in Figure 7a) for both OCS and MCS. Obviously, an increase in the elastic interaction strength, δ for OCS or ⟨δ⟩ for MCS, does not change the morphology of the adsorptive layer. In the case of elevated adsorption rate α = 0.06, even in the absence of elastic interactions δ = ⟨δ⟩ = 0 for both OCS and MCS, separated adsorbate islands emerge on the substrate (see the stability diagram in Figure 3 and the corresponding snapshot in Figure 7b). An increase in δ leads to the transformation of the morphology of the surface layer accompanied by the formation of elongated adsorbate islands (see snapshots at δ = ⟨δ⟩ = 0.2 and δ = ⟨δ⟩ = 0.4 in Figure 7b) for both OCS and MCS.

**Figure 8:**
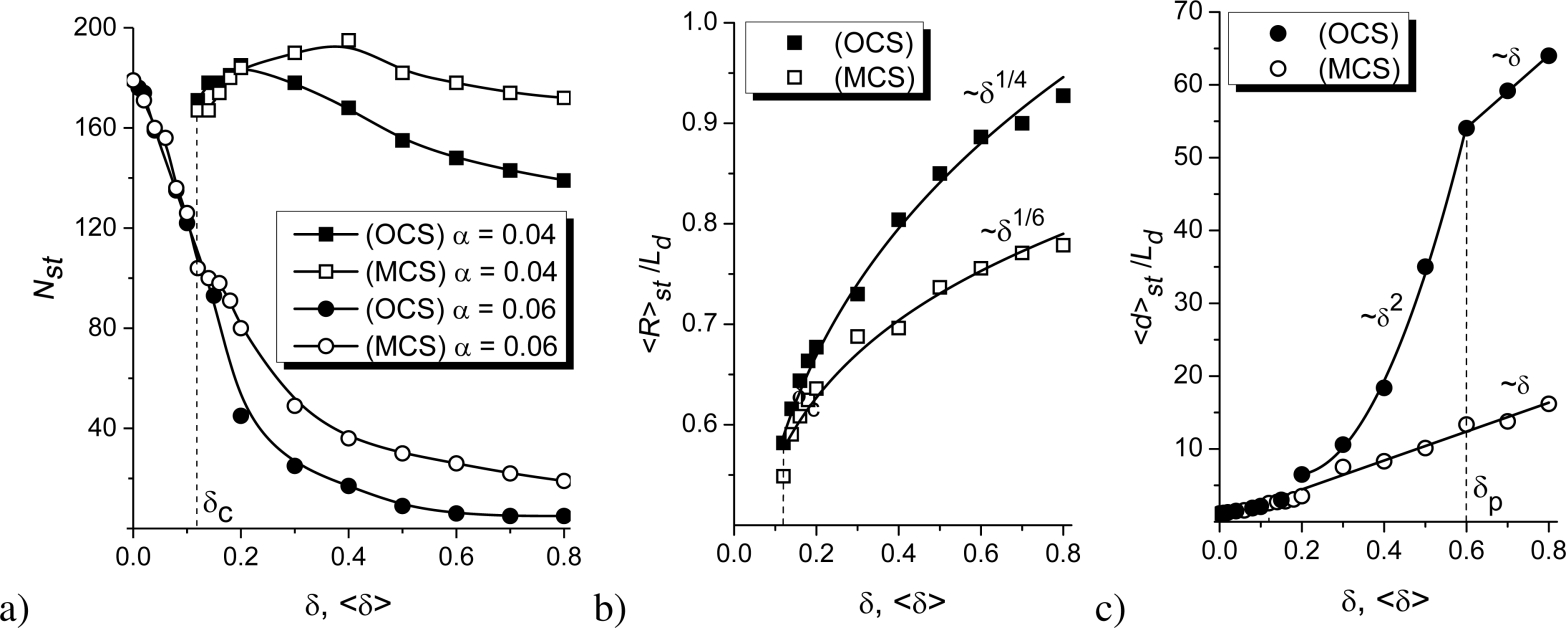
Dependencies of (a) the number *N_st_* of adsorbate islands at different α, (b) the mean radius ⟨*R*⟩*_st_* of spherical islands at α = 0.04, and (c) the length ⟨*d*⟩*_st_* of elongated structures at α = 0.06 on the substrate–adsorbate interaction strength δ at ε = 4.0 for the OCS (filled symbols) and the MCS (empty symbols).

Dependencies of the number of adsorbate islands and their mean size on the elastic interactions strength are shown in Figure 8. It follows that at low adsorption rate α = 0.04, an increase in δ results in a non-monotonic dependence of *N_st_*(δ) for both OCS and MCS (see Figure 8a). Here, after δ = δ*_c_*, the number of adsorbate structures increases, attains maximal value, and starts to decrease. Moreover, at small δ *>* δ*_c_*, the type of the substrate does not affect *N_st_*. At elevated δ, deposition on the MCS results in a larger amount of adsorbate islands than on OCS. In the case of elevated α, an increase in the elastic interaction strength leads to a morphological transformation associated with the formation of elongated structures (see Figure 7b). Under such deposition conditions, *N_st_*(δ) monotonically decreases with the growth of δ. The role of the substrate configuration remains the same as in the previous case, that is, at small δ, the structure of substrate has no essential effect; at elevated δ, the number of adsorbate structures realized on the MCS is larger than on the OCS.

Finally, let us discuss the influence of the interaction strength and the structure of the substrate on the mean size of adsorbate structures shown in Figure 8b,c. For the case of deposition at the low adsorption rate α = 0.04, the adsorbate islands remain spherical (see Figure 7a), and one can use a unique size, that is, the mean radius ⟨*R*⟩*_st_*, for their description. The dependencies ⟨*R*⟩*_st_*(δ) at α = 0.04 for both OCS and MCS are shown in Figure 8b. It follows that with increase in δ *>* δ*_c_*, the radius of spherical adsorbate islands increases for both types of substrate in power-law form ⟨*R*⟩*_st_*(δ) ∝ δ*^g^*. The power-law exponent *g* for the deposition on MCS is *g* = 1/6, that for the deposition on OSC is *g* = 1/4. Here, adsorbate islands are characterized by a larger mean size compared to the case of MCS at the same strength of the elastic adsorbate–substrate interaction strength δ.

In the case of the elevated α = 0.06 (see Figure 7b), we will use two sizes for description of the adsorbate structures, that is, the transverse size 2⟨*R*⟩*_st_* and the longitudinal size ⟨*d*⟩*_st_*, by associating the adsorbate islands with a rectangle with sides 2⟨*R*⟩*_st_* and ⟨*d*⟩*_st_*. In this case, we assume that the transverse size does not change crucially with increase in δ, whilst ⟨*d*⟩*_st_* increases with δ. By using ⟨*R*⟩*_st_*|*_δ=0_* at α = 0.06, we have computed the mean length ⟨*d*⟩*_st_* of elongated adsorbate structures for different values of the elastic interaction strength δ, shown in Figure 8c. It follows that for both types of substrate, the mean length increases with the growth of δ. At elevated δ, for the deposition on MCS ⟨*d*⟩*_st_* grows according to the linear law ⟨*d*⟩*_st_*(δ) ∝ δ. For the case of OCS, there are two different regimes, namely, the parabolic growth ⟨*d*⟩*_st_*(δ) ∝ δ^2^ at δ *<* δ*_p_* and the linear dependence ⟨*d*⟩*_st_*(δ) ∝ δ at δ *>* δ*_p_*. This critical value δ = δ*_p_* relates to the formation of percolating cluster of adsorbate connecting opposite sides of the substrate (see Figure 7b). The number of percolating clusters increases with growing δ.

In order to estimate the mean linear size of spherical adsorbate islands ⟨*R*⟩*_st_*, one can exploit the relation *L_d_* = 20*r*_0_ with *r*_0_ = 1 nm [49], which gives ⟨*R*⟩*_st_* ∈ (12,20) nm. This result corresponds well to experimentally observed small separated adsorbate islands of Al or Cu condensates on glass substrates [17–19].

## Conclusion

In this article, we have studied the effects of adsorbate–substrate interactions onto the morphology of growing thin films during low-pressure condensation in the framework of theoretical approach and numerical simulations. It is shown that an increase in the strength of adsorbate–substrate interactions results in first-order transitions from a low-density state toward a high-density state. It is found that at extremely low pressures of the gaseous phase, these interactions induce self-organization processes of adatoms leading to the formation of separated adsorbate islands on the substrate. The obtained theoretical results are verified by numerical simulations. In the framework of computational studies, we have discussed deposition on a one-component substrate and a multicomponent substrate representing high-entropy alloys. It is found that an increase in adsorbate–substrate interaction strength stimulates the formation of stable surface structures during deposition and leads to the formation of a smaller number of adsorbate clusters. During deposition on the disordered multicomponent substrate, the adsorbate layer is characterized by an increased amount of smaller islands compared to deposition on the one-component substrate. At elevated pressure inside the chamber, an increase in interaction strength between adsorbate and substrate results in the morphological transformation of the growing layer from separated adsorbate islands toward separate nanoholes inside an adsorbate matrix due to the percolating structure of the adsorbate.

Summarizing, one can conclude that the effects of adsorbate–substrate interactions during nanostructured thin film growth at low-pressure deposition are profound and multifaceted. Understanding and controlling these interactions are essential for tailoring thin film properties to meet specific application requirements. Further research efforts focusing on elucidating the underlying mechanisms of adsorbate–substrate interactions and their impact on thin film growth dynamics will continue to drive advancements in materials science and technology. These efforts are crucial for realizing the full potential of nanostructured thin films in enabling next-generation devices and technologies.

The general results obtained within this work can be useful to predict the morphology of the growing surface during low-pressure condensation and to select the technological conditions and appropriate materials for substrate and adsorbate to obtain nanostructured thin films with predetermined morphology and statistical properties of surface structures. The used dimensionless parameters adsorption rate and energies of adsorbate–adsorbate and adsorbate–substrate interactions, can be rescaled for particular adsorbate–substrate systems by exploiting data from the experimental studies and/or atomistic modeling.

## Data Availability

The data that supports the findings of this study is available from the corresponding author upon reasonable request.
